# Risk factors and preventive strategies for unintentionally retained surgical sharps: a systematic review

**DOI:** 10.1186/s13037-021-00297-3

**Published:** 2021-07-12

**Authors:** Samuel Weprin, Fabio Crocerossa, Dielle Meyer, Kaitlyn Maddra, David Valancy, Reginald Osardu, Hae Sung Kang, Robert H. Moore, Umberto Carbonara, Fernando J. Kim, Riccardo Autorino

**Affiliations:** 1 Division of Urology, Department of Surgery, VCU Health, Richmond, VA 23298-0118 USA; 2 Division of Urology, Magna Graecia University of Catanzaro, Catanzaro, Italy; 3 Dept of Urology, Andrology and Kidney Transplantation Unit, University of Bari, Bari, Italy; 4 Division of Urology Denver Health Medical Center and University of Colorado Anschutz Medical Center, Colorado Denver, USA

**Keywords:** Detection, Foreign object, Management, Retained surgical item, Surgical sharps

## Abstract

**Background:**

A retained surgical item (RSI) is defined as a *never-event* and can have drastic consequences on patient, provider, and hospital. However, despite increased efforts, RSI events remain the number one sentinel event each year. Hard foreign bodies (e.g. surgical sharps) have experienced a relative increase in total RSI events over the past decade. Despite this, there is a lack of literature directed towards this category of RSI event. Here we provide a systematic review that focuses on hard RSIs and their unique challenges, impact, and strategies for prevention and management.

**Methods:**

Multiple systematic reviews on hard RSI events were performed and reported using PRISMA (Preferred Reporting Items for Systematic Reviews and Meta-Analyses) and AMSTAR (Assessing the methodological quality of systematic reviews) guidelines. Database searches were limited to the last 10 years and included surgical “sharps,” a term encompassing needles, blades, instruments, wires, and fragments. Separate systematic review was performed for each subset of “sharps”. Reviewers applied reciprocal synthesis and refutational synthesis to summarize the evidence and create a qualitative overview.

**Results:**

Increased vigilance and improved counting are not enough to eliminate hard RSI events. The accurate reporting of all RSI events and near miss events is a critical step in determining ways to prevent RSI events. The implementation of new technologies, such as barcode or RFID labelling, has been shown to improve patient safety, patient outcomes, and to reduce costs associated with retained soft items, while magnetic retrieval devices, sharp detectors and computer-assisted detection systems appear to be promising tools for increasing the success of metallic RSI recovery.

**Conclusion:**

The entire healthcare system is negatively impacted by a RSI event. A proactive multimodal approach that focuses on improving team communication and institutional support system, standardizing reports and implementing new technologies is the most effective way to improve the management and prevention of RSI events.

## Background

The occurrence of a retained surgical item (RSI), also commonly known as the unintended retention of a foreign object (UFRO), is a rare but potentially serious event that has significant patient, physician, and hospital implications [1]. Intraoperative RSIs can be categorized as either soft (e.g. sponges, gauze, packing, towels) or hard (e.g. needles, blades, instruments, guidewires, or fragments) [1]. RSI events are believed to occur once in every 1000 to 18,000 surgeries [2–6], however this is likely an underestimation due to presumed underreporting and the exclusion of “near miss” events [5, 7–9]. In the general population, the rates of RSIs are highest in abdominal, gynecologic, vascular, and urologic procedures [2, 3, 10, 11] and have a variety of associated risks and clinical presentations. In the pediatric population, interventional radiology and neck procedures have the highest rates of RSI events [2]. RSIs continue to be the most frequently reported sentinel event to the Joint Commission (JC) since it started publishing data in 2012 [12, 13].

In the last two decades, many landmark reports and population-based studies have brought increased attention to patient safety as a national health care priority, acting as a stimulus for policy change [1]. In the operating room, increased vigilance has been directed towards prevention of near miss and never events with improved protocols and introduction of new technology to decrease the incidence of RSI events [14]. Despite new policies and procedures, incidents continue to occur and RSI events remain the number one sentinel event in 2019 with a relative rise in the incidence of hard foreign bodies compared to soft foreign bodies [13, 15].

Here we present a systematic review of the current literature on risk factors, prevention, clinical and economic impact, and detection of RSIs with a focus on the management and specific challenges related to hard RSI events.

## Materials and methods

**Fig. 1:**
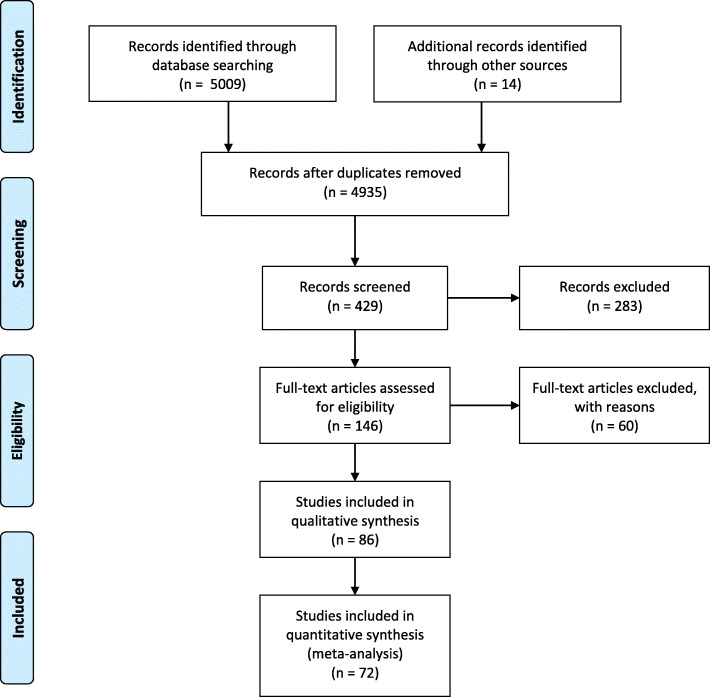
PRISMA flowchart

Multiple literature searches were performed according to the PRISMA methodology (Fig. 1). The International Prospective Register of Systematic Review (PROSPERO) protocol number is CRD42020218848. The objective was to assess incidences, challenges, risk factors, and strategies for prevention and management of hard RSI events, as well as the impact created by metallic RSIs (e.g. needles, blades, instruments, wires and fragments), more commonly referred to as “surgical sharps” on patients, providers, and health systems. All searches were restricted to the last 10 years and excluded ingested sharps, non-thoracic and abdominal sharps (e.g. ocular sharps) and retained foreign bodies from non-iatrogenic causes (e.g. trauma). Initial screening was conducted by article title and abstract. After identification of the relevant studies, full text review was performed. The reviewers then applied the principles of refutational synthesis and reciprocal translation analysis for conducting a qualitative meta-narrative review of the current literature.

## Results

A total of 72 articles were included in this qualitative synthesis. The true incidence of hard RSIs is difficult to ascertain, representing a significant challenge towards RSI incident reduction. Reporting is voluntary at many institutions, and physicians commonly forgo reporting RSI events either intentionally (due to fear of litigation) or inadvertently (because of a different interpretation of the incident) [14].

This is especially true for “near miss” events, where a significant amount of time is spent searching for a surgical item that is ultimately recovered or when a post-operative x-ray leads to the removal of a RSI. Near miss events are evaluated as a marker for patient safety and can offer valuable information not captured by adverse event reports [16]. In a large-scale institutional study reporting 191,000 operations over 4 years, an equal number of RSI events and near miss events were reported, with 76% of these near misses being attributed to a miscounted needle [17]. Due to the difficulty of detecting missing needles in the patient, these near miss events could very well represent an actual retained item [18]. Yet, despite their high incidence and marker status for patient safety, there remains a paucity of literature discussing operative near miss events – undoubtedly a missed opportunity for improvement of patient safety.

### Risk factors

Reports that discuss risk factors for RSI events rarely categorize by the type of retained foreign object. A previous meta-analysis of three retrospective case control studies found upon pooled analysis that there were seven factors significantly associated with an increased risk for an RSI event including blood loss greater than 500 cc, prolonged operative time, more than one sub-procedure, more than one surgical team, unexpected intra-operative findings, lack of surgical counts, and incorrect counts [19]. BMI was also found to be significantly associated with RSI risk, although this association varied amongst existing literature [19–27].

**Fig. 2:**
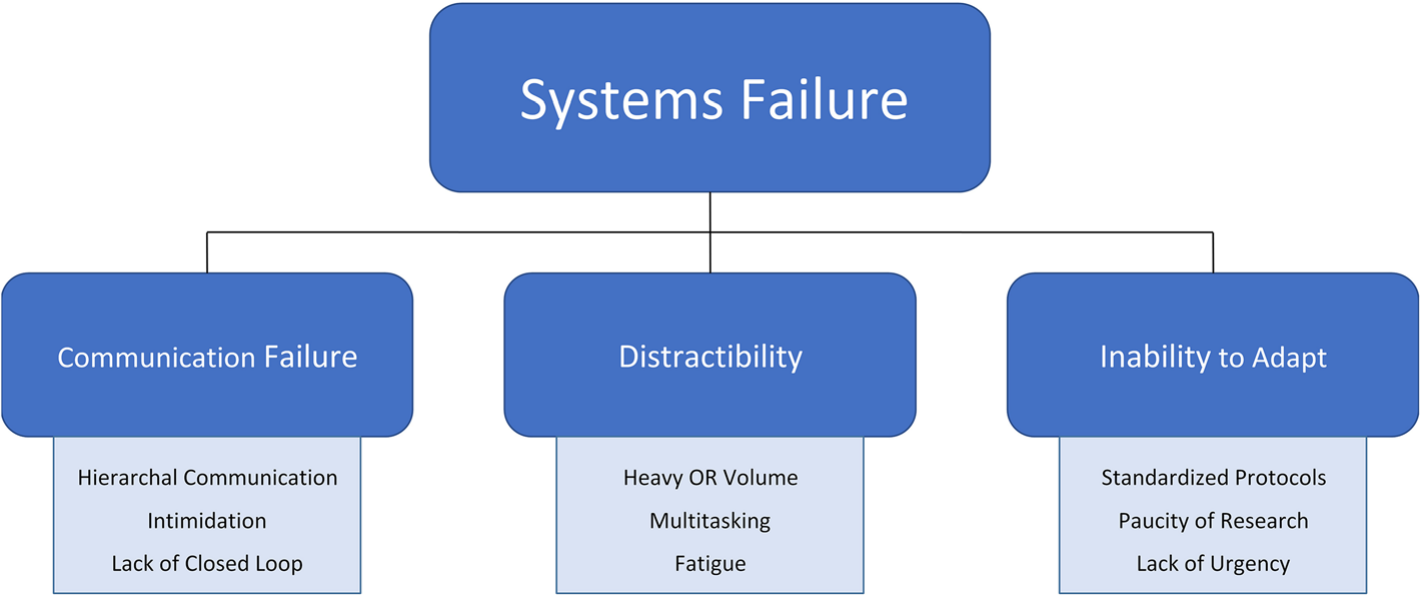
The importance of teamwork in RSI prevention (Figures can be printed in grayscale) Provenance and peer review not commissioned, externally peer-reviewed.

Most of risk factors identified for RSIs are dependent on how well the members of the surgical team work together (Fig. 2) [28]. The importance of teamwork (and related factors such as “communication failure”, “distractibility” and the lack of “adaptability”) was highlighted in one study where approximately 90% of RSI events were found to be secondary to team or system failures (as opposed to an error by an individual) [29]. Communication failures, occurring between surgical teams, surgeons and OR staff, surgeons and radiologists - are due to a wide variety of factors including issues with hierarchal communication, intimidation, and lack of closed loop communication and were all found to be associated with a high risk of a RSI.

Distractions frequently occur in the operating theater and come in many forms. Teams that use behavioral modification strategies, such as reducing multi-tasking amongst different operating room members, and using closed loop communication, can minimize distractions and thereby reduce RSI risk. The ability of operative teams to adapt to novel situations and rapidly change operative plans is intricately linked with improved communication and reduced distractions and therefore critical in reducing RSI events [28].

Although the literature varies, most agree that emergency operations and surgeries that require unexpected changes during the procedure are nine times more likely to result in a RSI event [30]. This is potentially explained by emergent situations occasionally necessitating deviation from safety protocols to save time.

Unexpected changes in surgical items and surgical team members make accurate counting and documentation more challenging and increase chances for communication failure between team members [2, 3, 9, 17, 20, 22, 24, 26, 30–32]. The JC has identified failure of communication, absence/non-compliance with RSI policies and intimidation resulting from hierarchal concerns amongst the surgical team as the main contributing factors for RSI events [12, 24, 29, 33, 34].

With the increasing use of minimally invasive surgical (MIS) techniques, there has been an expected increase in RSIs associated with MIS as compared to open procedures [12]. Risk factors associated with MIS include a limited field of view and the lack of tactile feedback for the operator [12, 17, 22] which make it more difficult to locate a lost object and therefore increase the risk of an RSI event [35, 36].

### Strategies for prevention and management

Different strategies and methodologies have been investigated for hard RSIs *(*Table 1***).***

**Table 1:** Strategies for prevention and management

METHOD TO PREVENT AND/OR MANAGE RSI	PROS	CONS	WAYS TO IMPROVE METHOD
**Manual** **counting**	RSI 100 times more likely if there is a count discrepancy [17] When combined with technological advancements, this can improve its accuracy [37, 38]	RSI can still occur with a correct count [39]	Quiet OR and reduce multi-tasking during count [40, 41] Change focus of count to RSI prevention rather than checklist [41]
**Intraoperative Radiography**	Quick and cheap [32, 42] Low radiation dose [32, 42] CT does not appear to be superior to intraoperative X-ray [4]	False negative rate increases as needle size becomes smaller [23, 43] needles smaller than 13 mm are not detectable [22, 34, 44] Dependent on radiologist’s knowledge of the lost needle and its last location [23, 45]	Development of standard policy of indications for radiography Education for all members of the team to improve communication between radiology and surgical team [31]
**Computer-aided Detection (CAD)**	Automatic detection rate as high as 86% [33, 46] Potential of faster and more cost-efficient solution than radiography [33, 46]	Currently in developmental phase [36] Unable to identify small needles [36] Relies on large dataset of needles and images to train the system [33, 36] Requires confirmation of results by surgeon and/or radiologist [6, 47]	Continued development of database and system
**Magnetic** **Retrievers**	Allows surgeon to follow metallic objects in real time, thereby expediting their removal [48] Reduces search time for small and medium sized needles [48]	Risk of injury to organs during retrieval [16, 48] Not FDA approved for sharps retrieval	Continued development to ensure patient safety.
**Sharps Finder** **Device**	Able to detect needles not visualized by x-ray [49] May act as a rule out mechanism preventing unnecessary radiation exposure. Expedites the identification of surgical sharps [49]	Only used to identify the location of a surgical sharp [49]	More clinical trials needed to determine degree of efficacy
**METHOD TO PREVENT AND/OR MANAGE RSI**	PROS	CONS	WAYS TO IMPROVE METHOD
**Manual** **counting**	RSI 100 times more likely if there is a count discrepancy [17] When combined with technological advancements, this can improve its accuracy [44, 45]	RSI can still occur with a correct count [39]	Quiet OR and reduce multi-tasking during count [37, 42] Change focus of count to RSI prevention rather than checklist [42]
**Intraoperative Radiography**	Quick and cheap [32, 47] Low radiation dose [32, 47] CT does not appear to be superior to intraoperative X-ray [4]	False negative rate increases as needle size becomes smaller [23, 49] needles smaller than 13 mm are not detectable [22, 34, 50] Dependent on radiologist’s knowledge of the lost needle and its last location [23, 41]	Development of standard policy of indications for radiography Education for all members of the team to improve communication between radiology and surgical team [31]
**Computer-aided Detection (CAD)**	Automatic detection rate as high as 86% [33, 51] Potential of faster and more cost-efficient solution than radiography [33, 51]	Currently in developmental phase [36] Unable to identify small needles [36] Relies on large dataset of needles and images to train the system [33, 36] Requires confirmation of results by surgeon and/or radiologist [6, 52]	Continued development of database and system
**Magnetic** **Retrievers**	Allows surgeon to follow metallic objects in real time, thereby expediting their removal [53] Reduces search time for small and medium sized needles [53]	Risk of injury to organs during retrieval [16, 53] Not FDA approved for sharps retrieval	Continued development to ensure patient safety.
**Sharps Finder** **Device**	Able to detect needles not visualized by x-ray [54] May act as a rule out mechanism preventing unnecessary radiation exposure. Expedites the identification of surgical sharps [54]	Only used to identify the location of a surgical sharp [54]	Need clinical trials to determine degree of efficacy

#### Counting

Manual counting has been the mainstay for prevention of hard RSIs, however, even with development of new counting techniques and protocols, counting discrepancies remain a common event [12, 39, 40, 55]. Studies report counting discrepancies occurring as often as 1 in every 8 cases, with sharps (typically needles) being the most miscounted item [7, 12, 30], followed by instruments and instrument fragments [17, 18, 55]. Incorrect manual counts are responsible for RSIs in approximately 62 to 88% of RSI events, and in approximately 20–50% of RSI events the surgeon proceeded with closing the patient even though at least one person was aware of a count discrepancy [3, 7, 56].

The time intensive nature of manual counting often leads to the manual counts being skipped [6, 22, 30, 41, 45, 56]. Interventions aimed at reducing distraction such as ensuring that the OR is quiet and the involved team members are not being asked to perform multiple tasks during counts could improve accuracy [50, 55].

Additionally, specific protocols for early recognition of damaged surgical instruments based on visual inspection before, during, and after each case have been shown to limit the risk of a RSI [37].

Recent advancements, such as barcoded counting systems and data-matrix labeled or RFID tagged instruments, have also been implemented to improve accuracy of manual counting [38, 52]. Unfortunately, each of these advancements are limited in their ability to count the most frequently miscounted surgical sharp – a needle. This problem could be addressed by the use of fluorescence needles. In one study, using UV fluoroscopy for retrieval, incorporation of fluorescent coated needles was shown to significantly improve the time it takes surgeons to recover lost needles compared to uncoated control needles in both laparoscopic and open procedures [42].

#### Radiography

Intraoperative radiography is the most frequently implemented technology to assist in the discovery of lost surgical sharps. Plain film radiography is the initial imaging modality of choice to detect most foreign bodies quickly and cheaply with relatively low radiation exposure [33, 51]. A recommendation from the JC that healthcare systems have a standard policy in place to prevent RSIs after a miscount has led to many centers creating protocols to establish when an intraoperative radiography is needed [43]. Some common criteria include emergency procedures, procedures that change from the scheduled procedure, surgery performed on obese patients, count discrepancy, or inability to verify the count [38, 43].

Intraoperative radiography has a reported false negative rate that ranges from 10 to 30%, which increases as the size of the RSI decreases [24, 53]. Furthermore, the likelihood of the radiologist successfully identifying the missing item on imaging is highly dependent on them knowing the detailed information about the type, expected appearance, and likely location of the missing object [24, 41]. This requires accurate and detailed bi-directional communication between the surgical team and the radiologist [21, 24, 43, 44, 54].

Several studies have shown that the ability to detect surgical needles is highly dependent on needle size. Detection is greatly reduced when needles are smaller than 17 mm, and unable to be identified on radiography if less than 13 mm [23, 35, 47].

A comprehensive quality assessment study comparing various radiographic techniques on minimal detectable needle size demonstrated that use of a mobile image intensifier is the most effective at detecting lost needles as compared to departmental radiography equipment and portable radiography machines [7].Another study evaluated whether CT was superior to radiography for detection of needles ranging in size from 4 mm to 90mm^5^. The authors found that both methods were comparable as far as identification of an RSI, however, CT was able to provide exact localization of the RSI with more specific landmarks to guide the search, while radiography was only able to give a general location [5]. Overall, it appears that there is no significant benefit in using CT over radiography, and that in the case of difficulties with localization, it can be solved with the use of other radiopaque structures to guide the search.

#### Computer-aided detection (CAD)

CAD software utilizes artificial intelligence with a modified map seeking circuit algorithms to identify RSIs automatically and is currently being developed to identify retained surgical needles with greater accuracy [53]. Using a CAD system while operating in high specific mode may allow surgeons to immediately identify and remove RSIs, offering a potentially faster, less susceptible to human error, and more cost-efficient solution than having a radiologist on call for the OR [7, 46]. Studies have shown great success with detecting micro-tagged sponges and to a lesser, but still significant extent, medium sized needles, with automatic detection rates reported as high as 86% [34, 48]. However, CAD systems are still in the early stages of development, and current CAD technology is unable to identify small retained needles [40]. The major limitations of the CAD system lie in the need to have a large dataset of reference image data for its training and that the results still require to be confirmed by the surgeon and/or a radiologist [34, 40].

#### Magnetic retrievers

In the event that a missing surgical item is identified on radiography, the challenge of locating and successfully removing the item must still be overcome. Needles and small instrument fragments are particularly difficult, as they can easily become buried in and around organs. The use of magnetic retrievers have been discussed to help expedite the removal of metallic surgical items, especially in minimally invasive procedures [17, 49]. Use of a magnetic retriever in a porcine model showed that both experienced and inexperienced surgeons were 11 times more likely to find a lost needle in less than 15 min when compared to a standard visual search, especially for small to medium sized needles [49]. While different magnetic retrieval devices are commercially available, widespread adoption remains a challenge due to limited availability [17, 49].

#### Sharps detector

To increase the ability to find surgical sharps not detectable via x-ray, new technologies have been developed. One example, the Melzi Sharps Finder®, exploits the small changes in the magnetic field associated with the presence of a surgical sharp to aid in identification [57]. The detector can be used laparoscopically or during an open procedure in a systematic search for a lost metallic sharp [10]. Unlike a magnetic retriever, however, the goal of this device is only to focus on identification rather than retrieval. At the time of this publication, the Melzi Sharps Finder® is registered with the FDA and undergoing clinical trials.

#### Systematic search

The Veterans Health Administration as well as the American College of Surgeons (ACS) have both recommended a thorough search of the body cavity or wound if there is a miscount of a surgical sharps [24]. In the setting of open surgery this is more straightforward when compared to minimally invasive surgery where the camera has limited visual scope and therefore a limited search area [49].

In a study based off a 15-question survey completed by 305 minimally invasive surgeons, Medina et al. proposed a systematic search protocol based on a primary visual search without the use of other instruments to reduce needle migration, which includes inspection of the port, the use of a magnetic retrieval device if available and intraoperative fluoroscopy, followed by a quadrant based systematic search with instruments, and inspection of the OR table, drapes, and floor [17]. These suggestions are supported by responses stating that the most common situations of needle loss are during insertion or removal of a needle through a port as well as laparoscopic suturing [29, 34, 53].

### Unsuccessful search for RSI

In the event where the lost item cannot be located using the previously discussed methods, surgical teams are faced with the difficult decision of when to stop searching. They must balance the possibility of a retained item remaining inside the patient with the risks of the search. For instance, during minimally invasive procedures, searching for and retrieving missing surgical items often leads to a conversion to an open procedure. However, some studies suggest that retrieval rates are not improved by conversion to an open procedure [58, 59]. Moreover, regardless of whether it is a minimally invasive procedure or an open procedure, the surgical team would be exposing the patient to increased time under anesthesia as well as the increased chances of injury to surrounding structures [58].

### Impact of RSIs

RSIs and near misses create the potential for a significant physical, psychological, and economic impact on the patient, physician, and hospital.

#### The patient

Searching for lost surgical items results in prolonged operative time, prolonged anesthesia, as well as increased risk of iatrogenic damage associated with the search and the use of intraoperative radiography [30, 60]. Patient’s response to RSIs depends on many factors, including the type and size of the retained item, duration in-situ, as well as the immune status and co-morbidities of the patient. Retained metallic items tend to be associated with an acute clinical presentation, while sterile sponges are more commonly associated with an insidious presentation [22, 29]. Local reactions to RSIs can be inflammatory and exudative resulting in abscesses, fistulas, obstructions, or erosions into nearby structures [24, 30, 46, 61–63]. Reactions can also be aseptic, forming stable granulomas, the progression of which can bring to compression on nearby structures, causing chronic pain and irritation or other more significant symptoms [10, 21, 22, 30, 35, 44, 61]. Complications of RSIs have been described in a variety of case reports, and it has been shown that patients with known RSIs are twice as likely to have at least one post-operative complication, with morbidity of approximately 50% [2, 30] and an extension of hospital stay in 59% up to 8 days [38].

In the case of an asymptomatic patient or an incidentally found RSI, one may decide to leave the foreign body in place and carefully observe for any potential complications. However, even in the best-case scenario when no medical complication arise, an RSI can still take a significant emotional toll on the patient, sometimes mandating psychotherapy for anxiety, and negatively affect the relationship with the surgeon and/or healthcare system [6, 22].

#### The physician

There is a growing awareness of the negative effect physicians experience from adverse surgical events, which has been termed the “second victim syndrome” [6, 64, 65].Because RSIs are considered never events according to the National Quality Forum, physicians can face significant professional reputation damage and even the risk of indefensible litigation [2, 36, 66]. This poses a psychological and emotional burden on the surgeon and contributes to reluctance to disclose RSI events [30]. The duration of the search for an RSI is also linked to physician harm. The longer it takes to search for a lost surgical item, the more stress a physician incurs which in turn impacts surgical team communication and is known to increase the likelihood of mistakes [12, 36, 67]. A recent cross-sectional survey of surgeons at multiple teaching hospitals in Boston revealed that intraoperative adverse events cause serious emotional distress in the 84% of the respondents. Anxiety, guilt, sadness, shame/embarrassment, and anger were the most frequently reported emotions, independent of the years of experience [64]. Yet, another survey demonstrated that residents have a greater risk of adverse consequences from their emotional distress, partly due to greater self-perceived responsibility and risk of repercussions [65].

When a lost surgical item is not retrieved at the time of the surgery, but identified later, surgeons face the challenging decision of whether to perform a repeat surgery to remove the RSI or observe. The data regarding the benefits of removing RSIs is limited, thus surgeons must carefully evaluate each incidence on a case-by-case basis. Intraoperative consults can be valuable to the operative surgeon, but the decision to ask for assistance is hindered by the reluctance to publicize their medical error. In a recent survey of minimally invasive surgeons, 64% noted experiencing at least one lost needle incident but only 14% reported their missing needle incidents, regardless of the outcome; furthermore, only 90% of health care professionals believe surgeons are always obligated to inform patients of missing and potentially retained surgical items [10].

In addition to concerns about their professional reputation, many surgeons are unmotivated to report surgical errors due to the risk of litigation exposure [68]. Physicians incur significant expenses with the cost of legal representation averaging approximately $30,000 in 2013, in addition to malpractice insurance ranging from $150,000 to $500,000. Malpractice suits vary, with a minimum of roughly $150,000 per patient in 2013 but can cost upwards of $5 million in certain cases [7, 12, 59].

#### The hospital

The costs associated with RSIs are vast and are not reimbursed by the Centers for Medicare and Medicaid Services. Hospitals absorb these additional costs in the form of extended length of hospital stays, prolonged operative time, use of x-rays, as well as repeat operations to remove RSIs. The addition of an RSI has been shown to nearly double the average cost of hospitalization and can prolong the operative time by as much as 1 hr. [3, 20, 36]

According to a publication by the JC in 2013, additional medical care due to an RSI is estimated to be approximately $70,000 per patient, and other studies have cited costs up to $200,000 [12, 24, 33, 59]. Various reports estimated medicolegal costs to be nearly $100,000 with a large settlement between $2 million to $5 million, regardless of the patient outcome [10, 17, 24, 69].

The second victim syndrome also carries potential financial consequences for the hospital systems; according to a 2004 review of physician turnover, replacing a physician that has left the surgical field due to burn out can cost hospital nearly $125,000 to recruit a prospective hire and $2,000,000 in lost revenue [65].

The significant liability for hospitals is further demonstrated by the lack of large RSI studies, especially ones that are not compiled from malpractice lawsuits’ data [20]. In one case-control study of multiple teaching hospitals, more than one institution refused to participate due to fear of exposing themselves to the risk of litigation, despite participation agreements guaranteeing absolute anonymity [3].

### Future directions

#### A cultural shift

Accurate reporting of all RSI events and near miss events is a critical step in determining ways to prevent RSI events and would allow for assessment of the efficacy of new technologies in identifying RSIs. The current lawsuit-motivated environment has created a barrier to transparency, which highlights the necessity for major systematic changes in the way that RSI events are handled [64]. Traditionally the blame of an RSI has been placed on the individual surgeon, however, over 90% of RSI events are the result of a team/system error [6, 29]. A proactive system’s approach to prevention of RSI should be adopted through continuous quality improvement with interprofessional teams participating in an in-depth review and careful scrutiny of the event without attributing blame [41, 70, 71]. By shifting the focus from assignment of blame towards identification of prevention strategies, a more transparent environment can be created [54]. Standardized protocols that involve the entire OR team will improve outcomes and encourage a shift towards a team-based mindset [6, 12, 54].

In addition, the surgical community needs increased awareness of second victim syndrome and its impact on the surgeon and hospital, with improvement in institutional support systems to help surgeons cope with RSI events. Programs such as confidential 24/7 physician counseling services, without disciplinary consequences, would be one strategy to improve the mental health of affected physicians.

#### Standardized reporting

The second greatest barrier to transparency that has been identified was the lack of standardization in reporting [64]. The implementation of a standardized reporting system must be done carefully to encourage reporting. Reporting systems must have a greater focus on fostering a supportive learning environment and solving safety issues, rather than being accusatory and hostile. This is especially important as medical errors are more often multifactorial rather than due to one individual’s failures, negligence, or incompetence [9, 64, 72, 73]. Disclosure of RSI to the patient’s is also critical to ensure that they are aware of possible sequalae which may require intervention in the future.

#### New technologies

The addition of new technologies, such as RFID sponge detection, has already proven beneficial in improving patient safety, patient outcomes, and reducing costs associated with retained sponges. Given the shift in the predominant type of RSIs from soft objects to hard [13, 15], new technologies that address hard retained foreign bodies must be explored to further reduce the total incidence of RSI events. Various identification and retrieval devices seem to be promising with increasing the success of metallic RSI retrieval [35, 59].

## Conclusions

The healthcare system is shifting towards a proactive rather than a reactive approach to medical errors. Continual reduction in the incidence of all RSI events will require improved preventative strategies as well as improved recovery strategies. RSI events are classified as “never events”, which suggests that they are completely preventable. However, their continued occurrence despite the development of many new protocols and regulations proves how complex and multifactorial the problem is. The current literature has a heavy focus on improving vigilance as well as historical methods of prevention. However, RSI event prevention requires a system-based solution that relies on the entire surgical team. Even then, human error and imperfections will always be present, which necessitates the implementation of technological support.

While technologies have proven beneficial for retained soft items thereby decreasing their rate of presentation, such successes have also exposed a gap in advancements regarding lost and retained hard items. This delay in the development of methodologies targeting metallic foreign bodies has contributed to their relative increase in the types of RSI events reported. Focus should be placed on developing technologies that improve detection of hard retained surgical items. By increasing the armamentarium used to identify and retrieve RSIs we can reduce the overall incidence of RSI events as opposed to changing the type of RSI that we see.

## Data Availability

Not applicatble.

## Abbreviations

RSS: Retained surgical sharp
NM: Near-miss sharp
PSC: Patient Safety Culture
OR: Operating Room
JC: Joint Commission
